# Systematic analysis, aggregation and visualisation of interaction fingerprints for molecular dynamics simulation data

**DOI:** 10.1186/s13321-024-00822-3

**Published:** 2024-03-12

**Authors:** Sabrina Jaeger-Honz, Karsten Klein, Falk Schreiber

**Affiliations:** 1 Department of Computer and Information Science, University of Konstanz, Universitätsstrasse 10, 78464 Constance, Germany; 2 Faculty of Information Technology, Monash University, Clayton, VIC 3800 Australia

**Keywords:** Interaction fingerprints, Molecular dynamics simulation, Microcystin, Aggregation, Visualisation

## Abstract

Computational methods such as molecular docking or molecular dynamics (MD) simulations have been developed to simulate and explore the interactions between biomolecules. However, the interactions obtained using these methods are difficult to analyse and evaluate. Interaction fingerprints (IFPs) have been proposed to derive interactions from static 3D coordinates and transform them into 1D bit vectors. More recently, the concept has been applied to derive IFPs from MD simulations, which adds a layer of complexity by adding the temporal motion and dynamics of a system. As a result, many IFPs are obtained from one MD simulation, resulting in a large number of individual IFPs that are difficult to analyse compared to IFPs derived from static 3D structures. *Scientific contribution*: We introduce a new method to systematically aggregate IFPs derived from MD simulation data. In addition, we propose visualisations to effectively analyse and compare IFPs derived from MD simulation data to account for the temporal evolution of interactions and to compare IFPs across different MD simulations. This has been implemented as a freely available Python library and can therefore be easily adopted by other researchers and to different MD simulation datasets.

**Supplementary Information:**

The online version contains supplementary material available at 10.1186/s13321-024-00822-3.

## Introduction

To understand and model 3D conformations and interactions crucial for the molecular recognition process and biological activity [1–3], different computational methods such as Molecular Dynamics (MD) simulations have been developed [4, 5]. These simulations produce long trajectories, which result in massive amount of time-dependent data and consists of individual atoms and their coordinates at specific time points. Currently, there are several bottlenecks such as computational speed or data analysis. When considering data analysis, as the size and length of the trajectories increase due to the increase in computing power, frame-by-frame analysis becomes more difficult and tedious. [6–8].

This is a particular bottleneck when trying to compare multiple simulations and highlight differences in i.e., interactions between simulations [9]. To identify interesting points in the trajectory, where e.g., changes occur, established measures that are commonly analysed and visualised include root-mean-square deviation, root-mean-square fluctuation (RMSF), radius of gyration and energy-based approaches [2, 10]. The identified time points or frames of interest are then often visually inspected by looking at the 3D conformations and interactions.

Systematically analysing and visualising the interactions derived from MD simulations is difficult. Different methods and tools have been proposed to aid in this process and, for example, to investigate interactions between a protein and ligand. Most of those methods are based on visual inspection (e.g., VMD [11]), visualisation of contact maps [12] (e.g., as a contact frequency map (MDContactCom [13]), or as dynamic matrix (CONAN [14])), or a list of interaction partners or distances (e.g., GROMACS [15] or MDAnalysis [16]). Even though different solutions have been proposed, they have certain disadvantages, such as difficulties in perceiving differences in multiple matrix visualisations, lists are complicated to analyse and lack 3D representation, and dynamic visualisation is difficult to remember as trajectories usually have many frames or time steps.

Recently, the concept of interaction fingerprints (IFPs) has also been proposed for MD simulations [17, 18]. They were originally designed to convert static 3D coordinates, such as those obtained from molecular modelling techniques or experimental studies, into a 1D bit vector [4, 5]. Several methods have been developed to derive IFPs of protein-ligand interactions, and most have been used as post-processing methods for virtual screening approaches (i.e., large-scale docking approaches) and conformational space analysis [4, 19–28] and have also been used for machine learning approaches [3, 29–31]. Unlike static structures, IFPs derived from MD simulations are more challenging to analyse because MD simulations allow studying the temporal motion and dynamics (e.g., conformation, interaction) of a system [1]. Therefore, many IFPs are derived from a single MD simulation [17, 18].

One of the first approaches to analyse IFPs of MD simulations was introduced by Kokh et al. [17]. A workflow was proposed to investigate ligand-protein interactions and calculate and analyse MD-IFPs for large systems of several hundred compounds. MD-IFPs developed in this approach were introduced to study unbinding (i.e., dissociation routes) and residence times in a trajectory and conformations of a series of compounds. MDAnalysis [16] and RDKit [32] were used and combined to read and iterate over MD simulation frames and to identify and compute interactions which were mapped to a bit vector. The resulting MD-IFPs were then mapped based on the ligand centre of mass on a 3D grid in either a physical or IFP space, and subsequently clustered with k-means or Gaussian methods. Transitions between the identified clusters were visualised and used to study intermediate states (meta-stable structures) relevant to dissociation. In addition, different matrix visualisations were proposed, which include either Euclidean distance between clusters, or a comparison of interactions between clusters [17].

**Fig. 1:**
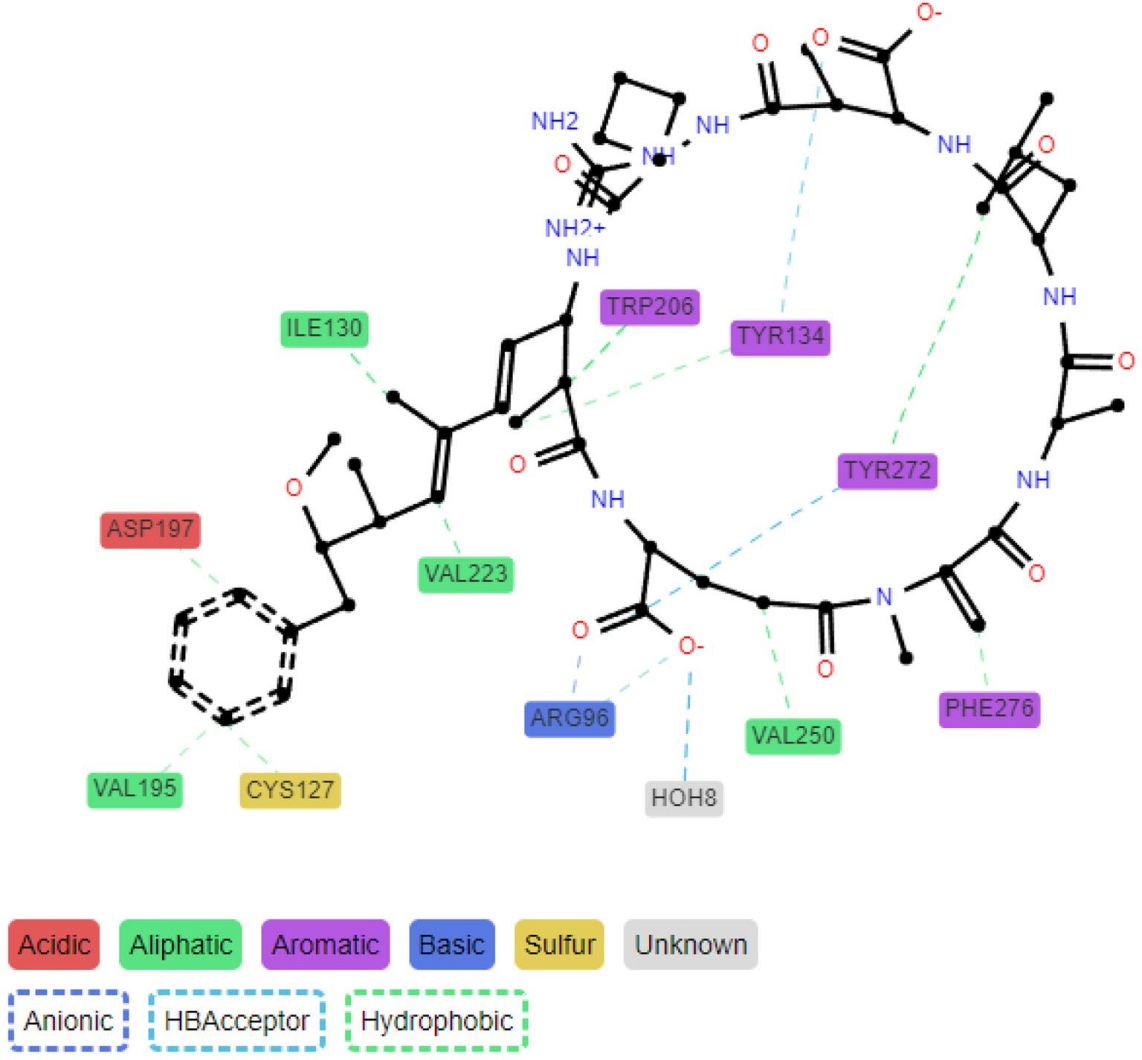
Aggregated frame with interactions occurring more than 30 % of the time calculated and visualised with ProLIF [18] (own data, PPP1-Microcystin-LR complex)

In 2021 a python library called ProLIF (Protein-Ligand Interaction Fingerprint) was proposed by Bouysset and Fiorucci [18]. ProLIF calculates IFPs from experimental data, docking poses or MD simulation data for a variety of molecules. It supports many different interaction types and additional ones can be added or edited by the user. Similar to the approach by Kokh et al. [17] MDAnalysis is combined with RDKit to analyse interactions either on residue or atomic level and results are provided as data frame for further processing and options for visualisation. Individual interactions are represented as a timeline, while for the analysis of all interactions, a so-called aggregated frame, is calculated. For the calculation of the aggregated frame, all interactions identified in the IFPs are summed up over time and interactions that occur more than 30% of the time are considered as present in the aggregated frame (see Fig. 1). The aggregated frame, or any specific frame at a specific time, can be interactively visualised at the atomic level for the ligand and at the residue level for the protein. The atom group highlighted on the ligand is the atom group most frequently interacting with the protein residues. In addition, a residue interaction network is provided, as well as a Tanimoto similarity matrix, which indicates the similarity of each MD simulation frame (or time point) to assess whether interactions (or IFPs), and therefore protein-ligand binding, change over time [18].

To systematically study and explore interactions that occur in large MD simulations, IFPs are a valuable tool as they are easy to handle as 1D bit vectors. Nevertheless, IFPs proposed for MD simulation data in previous work have the disadvantage of massively aggregating data by considering only frequently occurring interactions (i.e., more than 30% of the time) resulting in one representative IFP [18], or by loosing information after aggregation to clusters [17]. In addition to the one representative IFP, the ProLIF library, for example, also provides access to the individual IFPs corresponding to each frame in the MD simulation trajectory, which can be accessed and visualised as a network [18]. The advantage is that the user can select interesting IFPs from a particular frame of the simulation. The disadvantage is that it produces as many IFPs as the number of MD simulation frames analysed, and gives no indication to the user as to which IFPs might be of interest. For these reasons, the aim of this work was to develop a new method for the analysis and visualisation of IFPs derived from MD simulation data in order to systematically aggregate interactions and thereby reduce the number of IFPs, that is, the number of time frames. Furthermore, the developed methods facilitate the comparison of multiple simulations of the same system, which has been neglected so far.

## Application

### Data set

As a case study, we use the previously published MD simulations of PPP1 in complex with Microcystin congeners [33–36]. Microcystin (MC) congeners are a class of potent toxins released during cyanobacterial blooms worldwide [37]. They share a common overall cyclic structure [38] and can cause serious intoxications [39] and in extreme cases death [40–42]. The toxicodynamics inside the cell involves reversible and irreversible binding to PPP1, PPP2A, PPP5 and PPP6 [43–45]. Therefore, binding of MC congeners to PPP1 has been studied and analysed by Jaeger-Honz et al. [33]. Two simulations of MC-congeners, namely MC-LR and MC-LF, independently in complex with PPP1 were selected to analyse interactions. Since coordination via water molecules and manganese ions (Mn^2+^) is crucial for binding, these molecules have been included in the simulation [46]. Three replicates are available for each MC congener with a total length of 280 ns. After discarding the initial, non-equilibrated portion of the simulation, approximately 75,000 frames remain for analysis. [33].

### Interaction fingerprint calculation

To calculate IFPs from the MD simulation, ProLIF (v1.1.0) [18] was used with RDKit (v2021.03.5) [32] and MDAnalysis(v2.4.0) [16, 47] as described in the ProLIF tutorials.

Different interaction types are available for IFP calculation in ProLIF: Anionic, CationPi, Cationic, EdgeToFace, FaceToFace, HBAcceptor, HBDonor, Hydrophobic, Interaction, MetalAcceptor, MetalDonor, PiCation, PiStacking, XBAcceptor, XBDonor and VdWContact. X denotes halogen atoms. VdWContact was removed from the IFP calculation, because test runs showed that all interactions were changed to van der Waals contact rather than more specific ones. For Mn^2+^ VdWContact was analysed separately, as this interaction might be more unspecific. Mn^2+^ which are crucial for binding do not have a van der Waals radius assigned in MDAnalysis which is necessary for interaction calculation. Therefore, the parameters of magnesium ions were assigned as they have a similar size and coordination preference compared to Mn^2+^ [48] and were also used for the simulations used here (see Jaeger-Honz et al. [33]). The IFPs were calculated for all frames in our MD simulation data, and replicates were treated as a single entity. Therefore, approximately 75,000 IFPs could be obtained for each MC congener.

## Implementation

**Fig. 2:**
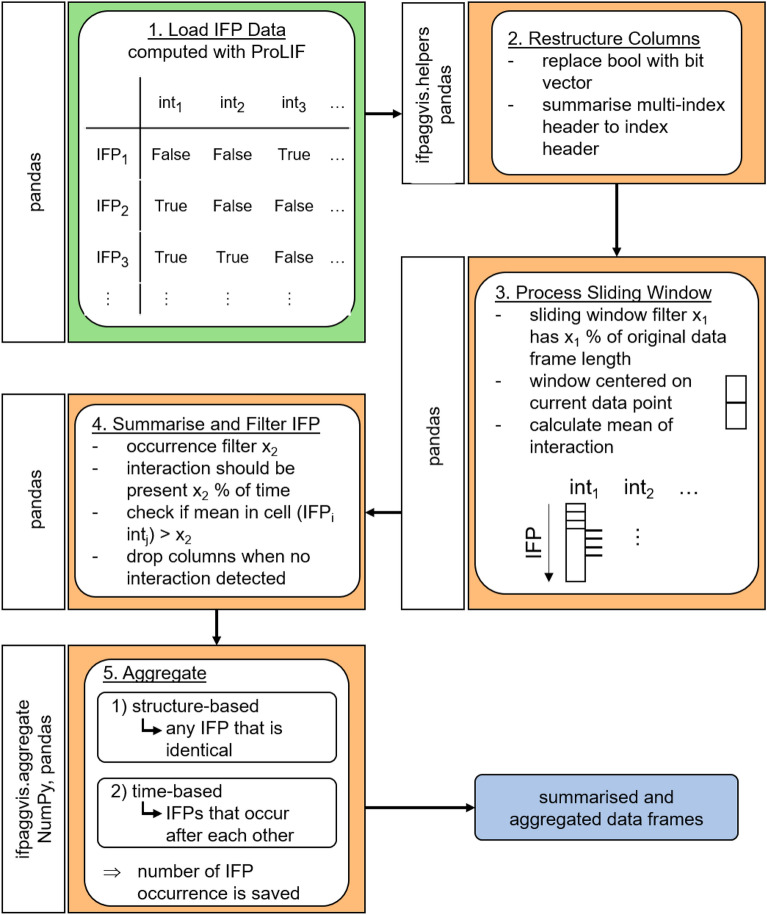
Flow chart of pre-processing and aggregation of IFP data frame derived from MD simulation. int_1_, int_2_ etc. stands for interaction 1, interaction 2 etc. The Python packages used in each step are shown in the rotated white boxes

**Fig. 3:**
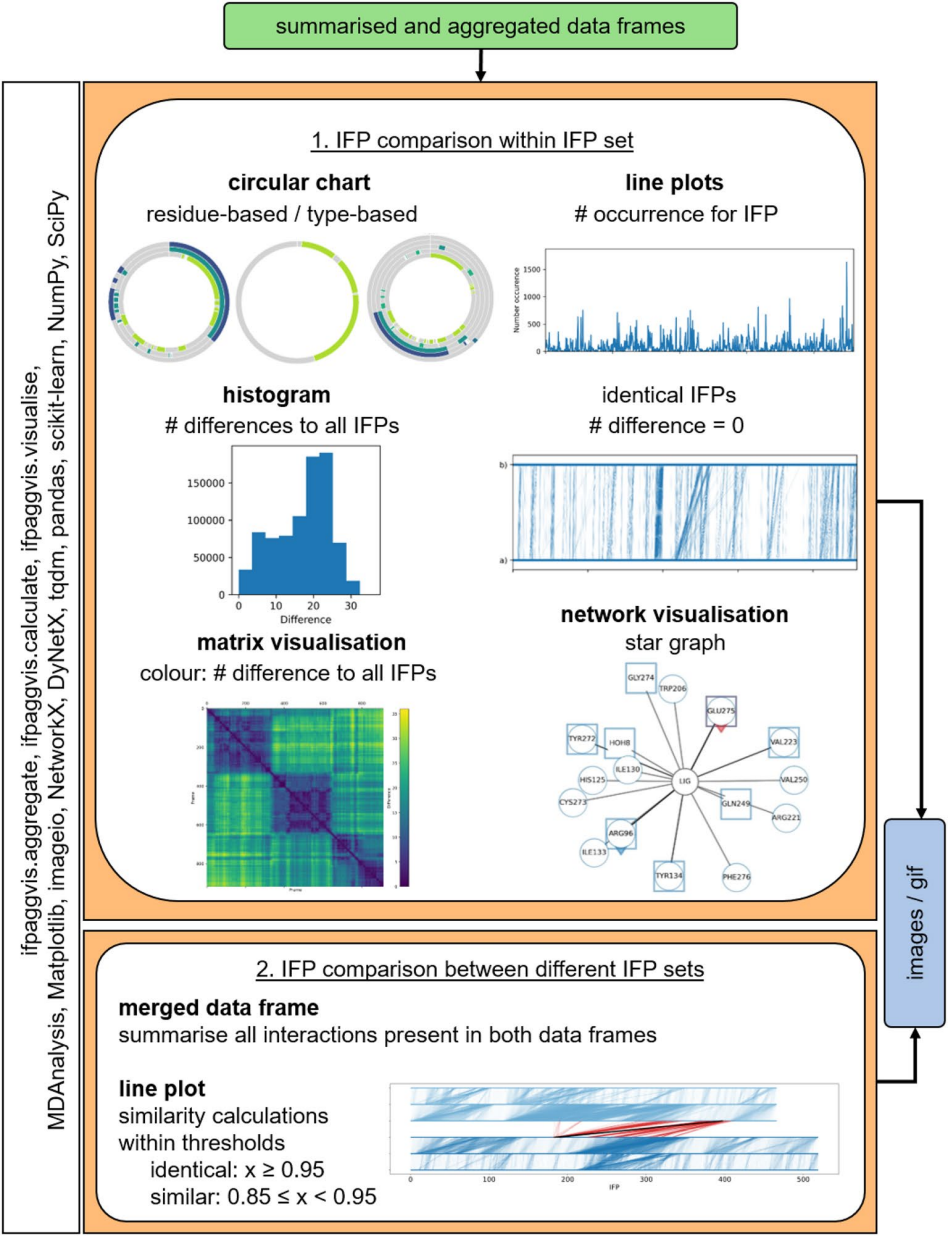
Flow chart of visualisations developed to compare IFP sets within and in between MD simulation. The Python packages used are shown in the rotated white boxes

We here present IFPAggVis, which is a Python library to aggregate, visualise and compare IFPs of MD simulation data. There are two major steps:

1. Pre-processing: IFPs are modified to summarise relevant interactions, and aggregated based on interaction or time (see orange boxes in Fig. 2),
2. Visualisation and comparison: Visualisation of similarity within IFPs of the same simulation and in between simulations are compared, evaluated, and visually assessed (see Fig. 3).

IFPAggVis is designed to work with pre-processed data frames of IFPs (e.g., computed with ProLIF). The following libraries were used for implementation: MDAnalysis (v2.4.0) [16, 47], RDKit (v2021.03.5) [32], ProLIF (v1.0.0 [18]), NumPy (v1.21.2) [49], tqdm (v4.62.3) [50], pandas (v1.3.3) [51], scikit-learn (v1.0) [52], Matplotlib (v3.4.3) [53], imageio (v2.28.0) [54], Networkx (v2.6.3) [55] and DyNetx (v0.3.1) [56].

In the following, the individual steps of pre-processing and visualisation, as well as comparison of IFPs are summarised.

### Pre-processing of interaction fingerprints

The individual steps of the pre-processing pipeline are shown in Fig. 2 (see orange boxes) and summarised as follows:

1. Load data frame of IFPs which were pre-calculated with ProLIF (see “Interaction fingerprint calculation” section and green box in Fig. 2).
2. Restructure the data frame to resolve the multi-index generated by ProLIF and map the Boolean values (True/False) to a bit vector (1/0) to indicate presence or absence of interactions
3. Processing of the sliding window. To aggregate interactions, a sliding window is calculated over each interaction (i.e., columns) with pandas. To determine the size of the sliding window, x_1_ is calculated which is a percentage value based on the trajectory length (i.e., number of IFPs). The sliding window is centred around the currently calculated data points to consider interactions close in time together. The value assigned to the current data point is the mean value across the window.
4. Filtering of the calculated mean based on x_2_ (x_2_ sets the interaction to present (1) or absent (0) if a mean value is greater than x_2_). The variable x_2_ is based on the percentage of occurrence within a sliding window.
5. Aggregation of processed IFPs. The derived IFPs are aggregated based on two different approaches: 1) interaction-based where any identical IFPs independent of the temporal dimension is summarised, and 2) time-based where identical IFPs which occur immediately after each other are summarised. If IFPs are summarised, the number of IFPs summarised are saved.

The filtering based on x_1_ and x_2_ smooths the data of the retrieved IFP. While the x_1_ filter evaluates the occurrence of interactions within a time window, x_2_ considers only frequently occurring interactions which occur more often than a threshold within the sliding window. Since numerical simulation data has a limited accuracy and calculation errors occur, and interactions can occur very rarely within a very small-time window, they are filtered out using the x_1_ and x_2_ filters. As the x_1_ and x_2_ filter are dependent on the MD simulation and probably also the data set studied, both filters of x_1_ and x_2_ can be adjusted by the user. To investigate effects of different parameters of x_1_ and x_2_, different thresholds were studied. For x_1_ we evaluated: 0.5 %, 1 %, 1.5 %, 2 %, 2.5 %, 5 %, 7.5 % and 10 %; for x_2_ the calculated mean values were filtered based on 0.00, 0.01, 0.02, 0.025, 0.05, 0.10, 0.15, 0.20, 0.25, 0.30, 0.35, and 0.40. Because the mean values range between 0 and 1, the values correspond as well to percentage of occurrence of an interaction within the window. For this reason, the x_2_ filter is also referred to as a percentage in this paper for ease of reading. The percentage values evaluated were chosen to cover a wide range of different values to appropriately evaluate, as they are likely to depend on the data set and simulation settings. Both filtering values have been limited at the upper end, as data smoothing is already high at these percentage values and unlikely to provide meaningful results. In addition, for the smaller percentage values the steps chosen were smaller, since smaller values are considered to be more sensitive to changes than larger ones. The aggregation based on interactions and time has different advantages and disadvantages. The interaction-based filtering results in a collection of unique IFPs through the simulation and therefore the lowest number of IFPs without further aggregation, but the temporal evolution of interactions is lost. The time-based filtering on the other hand preserves the temporal component but may lead to duplicates of IFPs as states could be revisited and therefore result in a higher number of IFPs. For these reasons, both aggregation methods have been considered in the workflow with IFPAggVis so that the user can change the aggregation type and different thresholds. The processed, filtered and aggregated IFPs are provided to the user as Pandas DataFrame, which can be accessed computationally and saved to files or used for further downstream processing.

### Visualisation and comparison of interaction fingerprints

To compare and assess the similarity of IFPs within a MD simulation, the number of absolute differences was calculated (see Eq. 1).

1 $$\begin{aligned} N_{Diff}= \sum \limits _{i=0}^{n-1} | IFP 1_{i} - IFP 2_{i} | \end{aligned}$$

To compare and evaluate similarity of IFPs of different MD simulations, the Rogers-Tanimoto dissimilarity metric was computed as implemented in scikit-learn (v1.0) [52] and SciPy distance functions (v1.7.1) [57] as they are optimised for efficient calculation on large amount of data.

The Rogers-Tanimoto dissimilarity is defined in Eq. 2, where $c_{ij}$ is the number of occurrences in two 1-D vectors at position *i* and *j*, $c_{TT}$ is the number of bits set on (1, interaction present) in both vectors, $c_{FF}$ is the number of bits set off (0, interaction absent) in both vectors, and $c_{TF}$ and $c_{FT}$ is the number of bits set on in the first vector and off in the second vector and vice versa. For IFP comparison, the dissimilarity is a value between 0 (similar) and 1 (dissimilar).

The similarity of two IFPs is calculated as shown in Eq. 3. Up to now, the Tanimoto coefficient has mostly been used to evaluate the similarity of molecules or IFPs. However, a study by Racz at al. [58] has shown that there are other coefficients that produce consistent results on different benchmark datasets and are viable alternatives to the Tanimoto coefficient, i.e., the Rogers Tanimoto. Therefore, this metric was selected in this work because of the possibility for fast computation but can be exchanged with other similarity or dissimilarity metrics as offered by the Python libraries scikit-learn [52] or SciPy [57] for pairwise distance calculation.

2 $$\begin{aligned}&Dissim._{Rogers-Tanimoto} \nonumber \\&\quad =\frac{2 \times (c_{TF}+c_{FT})}{c_{TT}+c_{FF}+ 2 \times (c_{TF}+c_{FT})} \end{aligned}$$

3 $$\begin{aligned}&Similarity = 1 - Dissim._{Rogers-Tanimoto} \end{aligned}$$

The number of differences as well as the similarity calculations are available to the user as a NumPy array and can be used for further downstream processing.

Different visualisations were proposed to support analysis and comparisons of IFPs (see Fig. 3), because it is not possible to cover all aspects of IFPs relevant for analysis and comparison with a single visualisation. IFPAggVis partly provides the visualisation as summarised visualisations. The proposed combinations show different aspects, which together should aid in analysing and understanding the aspects of IFPs derived from simulation.

For visualisation and comparison, two different approaches are available: 1) within the same MD simulation, and 2) between two different MD simulations. For both approaches, the pre-processed and aggregated IFPs are used as input (see green box, Fig. 3). To compare IFPs within the same simulation, circular charts, line plots, histograms, a similarity matrix and a network visualisation were developed (see orange box, Fig. 3).

The visualisation of interactions as circular chart summarise each residue individually with all interaction types occurring. The circular chart gives an overview of the interaction length and makes it easier to compare which interactions appear and disappear over time, or are constantly present or absent over periods of time.

The number of differences between IFPs are visualised as a histogram and as a matrix visualisation. The histogram shows the distribution of the number of differences, and therefore how similar all IFPs are to each other. In the matrix visualisation, this is colour-coded using the viridis colour map, which is perceptually uniform and robust to colour blindness. The matrix gives an impression on the development of differences between the IFPs over the course of the simulation, and can keep the temporal information or individual frames.

Two different line plots are available. One line plot shows the number of occurrence of an individual aggregated frame, that is, how many IFPs have been aggregated structure-based or time-based to the respective IFP. The second line plot links identical IFPs (i.e., the number of difference is zero) within an MD simulation with a vertical line. The frame numbers are shown by two horizontal lines.

The interactions between a ligand and protein are shown as star graphs, where the ligand is in the centre and residues are arranged around it. Initially calculated x, y coordinates can be saved to a file for reuse in further visualisations. The different interaction types that are analysed with ProLIF are encoded with different glyphs and are summarised in Table 1. In addition to the network visualisation, a line plot is provided showing the number of occurrence of an individual IFP as well as its index to give the user an estimate of the occurrence of individual networks in the data set.

**Table 1:** Glyphs and colours used to encode different interactions as network with a ligand

Interaction	Glyph	Colour
Hydrophobic	Circle	Blue
HBAcceptor	Square	Blue
HBDonor	Square	Red
Anionic	Arrow down	Blue
Cationic	Arrow down	Red
CationPi	Arrow left	Red
PiCation	Arrow left	Blue
PiStacking	Arrow up	Blue
EdgeToFace	Arrow right	Red
FaceToFace	Arrow right	Blue
MetalAcceptor	Tick up	Red
MetalDonor	Tick up	Blue
XBAcceptor	Tick down	Red
XBDonor	Tick down	Blue
VdWContact	Circle	Red

For comparison of IFPs of two different simulations (see lower orange box, Fig. 3), further processing of the aggregated IFP sets is necessary, as some interactions may be unique to one of the simulations. Therefore, a so-called merged IFP set is built out of the two original aggregated IFP sets. All detected interactions are added as columns and if not previously present, the interaction is considered absent. To quantify and compare differences between IFPs of two simulations, similarity or dissimilarity of IFPs has to be evaluated. The comparison of two different IFP sets leads to a higher difference between the individual IFPs, therefore comparing the number of differences was not considered appropriate any more. For this reason, the Rogers-Tanimoto dissimilarity was calculated and converted into similarity (as shown in Eqs. 2 and 3).

Three different classes have been chosen to categorise IFPs to assess similarity: identical, similar and dissimilar. Similarity of fingerprints is a fuzzy concept and also data set dependent [59–63]. For molecular similarity, different thresholds have been suggested. In some papers, a Tanimoto coefficient (*Tc*) of $Tc > 0.85$ is considered as structurally similar [62, 64], others consider a $Tc > 0.5$ as similar, and $Tc \le 0.5$ as dissimilar [65]. Thresholds for IFPs for MD simulations have not been systematically evaluated, and exact thresholds are likely to also depend on the data set.

Based on visual inspection of the IFP sets derived from the MD simulation, we decided to set the threshold for similarity of $T_{c} \ge 0.95$ as identical, $0.85 \le T_{c} < 0.95$ as similar and $T_{c}  < 0.5$ as dissimilar. The thresholds can be adjusted by the user dependent on the data set studied. The IFPs classified as identical, similar and dissimilar are returned as a dictionary for each class and can be saved with Pickle to file.

To compare the IFPs of two different sets, a line plot was developed to evaluate similarity within and in between simulations. Each IFP set (i.e., MD simulation set) is represented by three lines: 1) as dark blue lines (a, b, c) and 2) as bright blue lines (d, e, f) with IFP number on x-axis. Identical IFPs within the same MD simulation are shown between a and b, and e and f. Identical and similar IFPs between simulations are visualised between c and d, and they are shown in black and red, respectively.

All introduced visualisations are returned as Matplotlib figure, which can be saved to file. The network visualisations are saved as image files or GIFs due to the large number of figures generated.

## Results and discussion

In the following, the MD simulations are referred to by MC congener name instead of PPP1-MC congener as in Jaeger-Honz et al. [33] for easier readability. The aggregated frame derived based on the ProLIF paper (with occurrence more than 30%) is referred to as aggregated_occ30_ IFP to distinguish from interaction- and time-based aggregation. Key findings are briefly summarised: Filtering and aggregation of IFPs 1) massively reduces their number, 2) helps to identify important residues of major representatives, 3) retrieves interactions known from the literature that never occur simultaneously in an IFP, and 4) aids in comparing IFPs across MD simulation sets to assess similarity of binding patterns.

### Aggregation and filtering of interaction fingerprints

The influence of pre-processing was investigated by calculating the number of interactions and IFPs retrieved. First, the aggregation by interaction or time is compared to the aggregated_occ30_ IFP, and second, the effect of applying different filtering options with aggregation by interaction or time was investigated. For an explanation on the filtering and aggregation options, see “Pre-processing of interaction fingerprints” section.

By generating and analysing IFPs of different MC congeners simulation without further processing with IFPAggVis, 86 interactions could be detected for MC-LR, and 55 for MC-LF. The number of detected interactions in the aggregated_occ30_ frame drops to 14 and 20, for MC-LR and MC-LF, respectively. Therefore, we conclude that the aggregated_occ30_ frame provides a good overview of the major interactions, but looses a lot of information by aggregating to one IFP.

The number of IFPs after aggregation by interaction or time varies dependent on the simulation and aggregation type. In the original data set approximately 75,000 IFPs could be retrieved. When aggregating the IFPs by interaction, the number of IFPs could be reduced to 36.7% (27529) for MC-LR and 30.7% (23009) for MC-LF, which is still too many to analyse visually. In comparison, the aggregation by time reduces the number of IFPs to 95.7% (71764) for MC-LR and to 95.5% (71613) for MC-LF, which is almost as many as retrieved without aggregation.

**Fig. 4:**
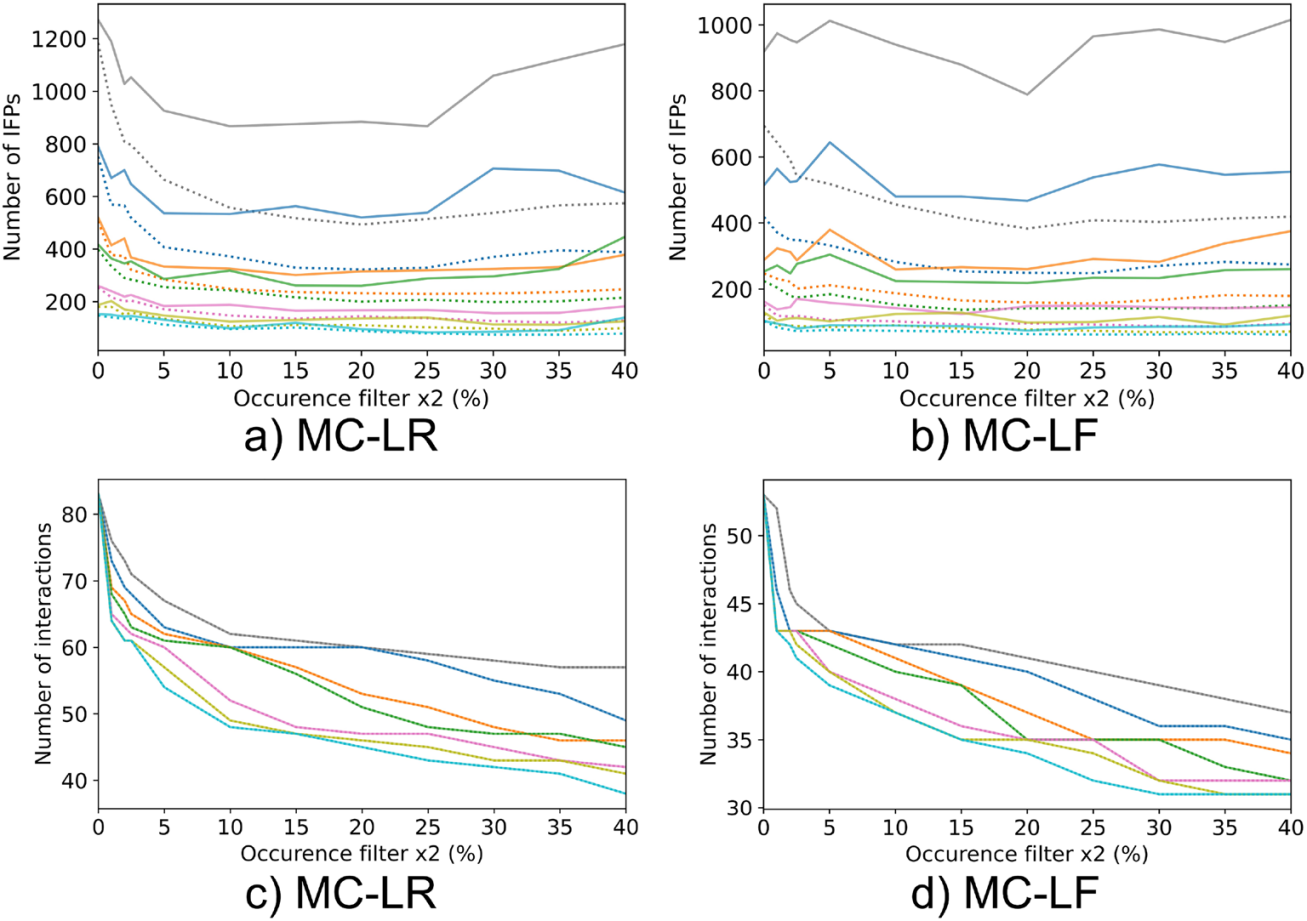
Number of IFPs (**a**, **b**) and interactions (**c**, **d**) after x_1_ and x_2_ filters are applied on MC-LR and MC-LF dataset. The x_1_ filters are coloured by value: 0.5% is grey, 1% is blue, 2% is orange, 2.5% is green, 5% is pink, 7.5% is yellow and 10% is cyan. The x-axis shows the x_2_ filter values. Solid and dashed lines represent aggregation based on time and interaction, respectively. The number of interactions is not affected by aggregation. Therefore, both lines are superimposed

The x_1_ filter (sliding window) massively reduces the number of IFPs (see Fig. 4 a and b). For the interaction-based aggregation, the number of IFPs is lower, as states can be revisited if aggregated by time. Independent of the size of the window chosen, less than 2% of the original number of IFPs remain. Smaller window sizes (0.5% and 1%) result in a higher difference between structural and temporal aggregation, which gradually disappears for larger window sizes (2%, 2.5%, 5%, 7.5% and 10%). For some sliding window filter sizes, a U-shaped curve is obtained (see Fig. 4 a and b). The curve shape is dependent on: 1) the aggregation type, as this effect is smaller with interaction-based aggregation, 2) the size of the sliding window as larger window sizes average out variance that may be present, and 3) the MC congener or the simulation analysed. This result seems to be counterintuitive at first. However, the number of IFPs left is not necessarily reflected by the filtering of occurrences. For some filtering options, the mean value before x_2_ filter is close to the selected x_2_ filter. Therefore, a change in threshold can lead to generation of several new IFPs by fluctuating absent and present interactions.

The number of interactions (see Fig. 4 c and d) is quickly reduced by using small x_2_ filter (percentage of occurrence) values (0 to 2.5%). Those interactions rarely occur within a small-time window and are probably not relevant, as they only exist for a short time span in the simulation and might be an artefact. Increasing the x_2_ filter above 20% does not lead to much further reduction of the number of interactions.

The filtering and aggregation settings for further IFP comparison and visualisation on our data set is therefore:

- Temporal aggregation: Revisitation of IFPs is considered important. Aggregation by interaction does not result in a significantly lower number of IFPs and is therefore not used.
- x_1_ filter (sliding filter) of 1% and x_2_ filter (occurrence filter) of 20%: The number of interactions decreases rapidly for a x_1_ filter below 1%, probably due to noise in the data. Window sizes of 2% and 2.5% show little difference to 1%. Larger window sizes than 5% have a high level of data smoothing, as interaction- and time based aggregation do almost not differ in the number of interactions. The 1% window on this dataset is approximately 7.5ns. With an x_2_ filter of 20 % an interaction is detected as present, if it occurred approximately 1.5 ns in the simulation, which is roughly the timescale where side-chain rotation and fluctuation occurs ($10^{-9}$s) [66]. This is considered as biologically appropriate, since shorter time scales are less relevant to interaction. In addition, the number of IFP and interactions retrieved between 10% and 20% are relatively constant.

### Analysis of interaction fingerprints of molecular dynamics simulation

#### IFP comparison within MC congeners simulation

**Fig. 5:**
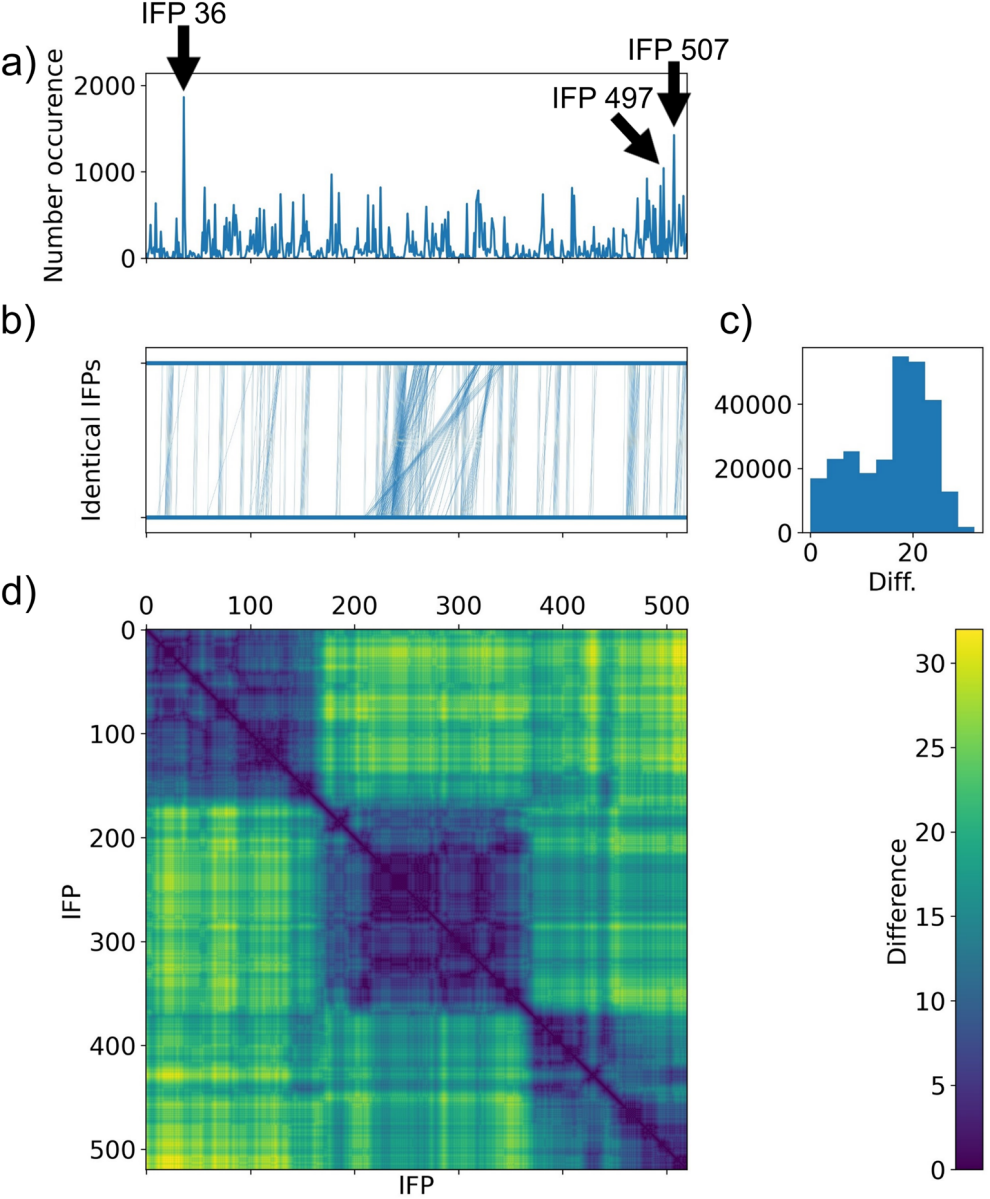
Comparison of IFP similarity within MC-LR. **a** Occurrence of each IFP with the three most frequent IFPs marked with arrows, **b** line plot connecting identical IFPs with vertical lines. The number of differences is shown as **c** histogram and **d** matrix with colour

The visualisations of filtered and aggregated IFPs of **MC-LR** and **MC-LF** MD simulation with PPP1 (see Fig. 5 and Additional file 1: Fig. S1a) show similar trends. Both matrix visualisations have areas of higher similarity indicated by large blue squares, which are divided into smaller nested squares that indicate regions of high similarity with a low number of differences. The number of differences increase over time (see yellow areas for distant IFPs), therefore changes accumulate over time. Identical IFPs are close together in time, which is visualised as vertical connections in the line plot above the matrix. The histogram visualising the total number of differences has two peaks: 1) small, around 10 differences, and 2) around 20 differences, indicating a group of IFPs that are close to each other but distant from others. The line plot with number of occurrence of individual IFPs show that there are major representatives that occur frequently.

**Fig. 6:**
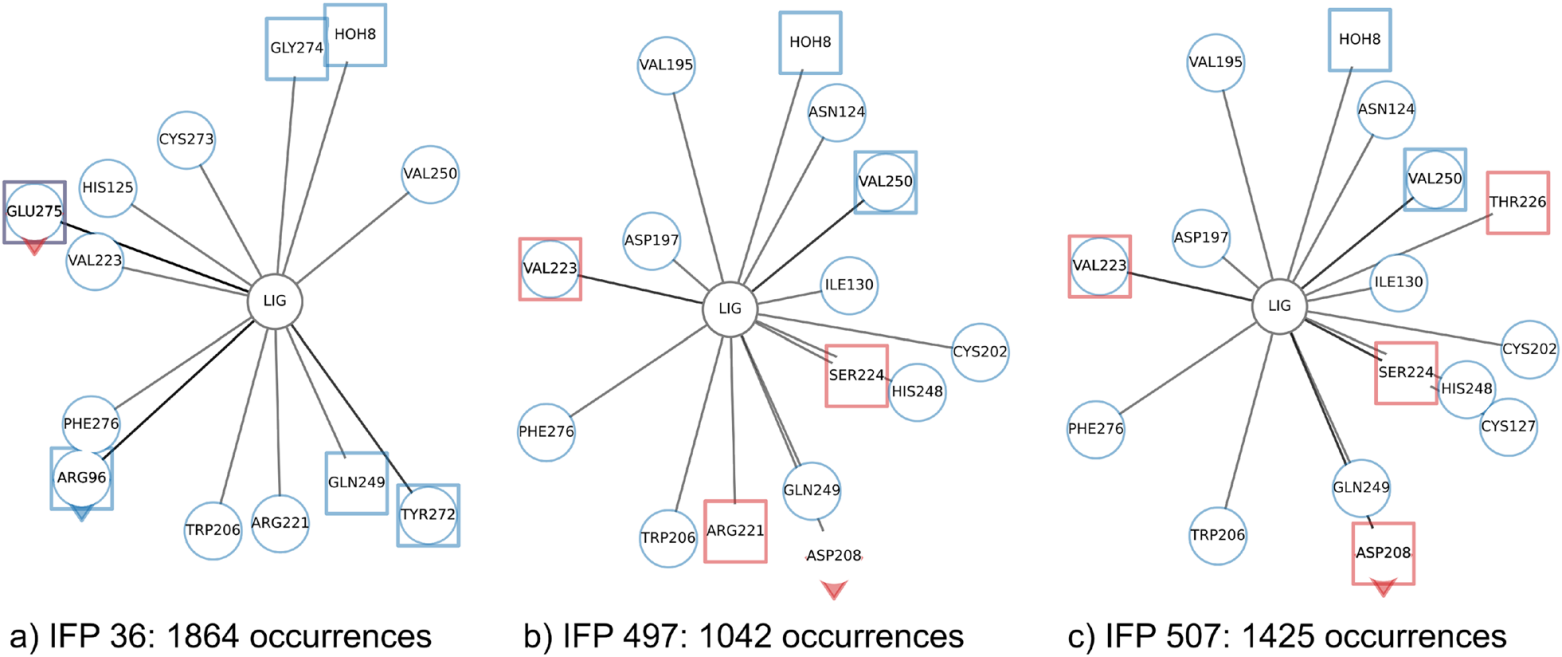
Comparison of the three most frequently occurring IFPs within MC-LR simulation

The residues important for interaction between MC congeners and PPP1 have been reviewed and summarised by Fontanillo and Köhn [46]. In brief the following interactions are important: 1) hydrogen bonds form with Arg96, Tyr134 and water to enable indirect coordination to manganese ions, 2) interaction with water is replaced by interaction between MC congener and Asn124, His125, Ile130, Tyr134 and Trp206, 3) hydrophobic interactions with Cys127, Ile130, Ile133, Trp206, Tyr272 and Gly274, and 4) a covalent bond which can be formed with Cys273 which is irrelevant here as bonds are not broken or formed with classical MD simulations as described here. Since different residues can interact in multiple ways with MC congeners, one residue is not restricted to one interaction. The three most common IFPs for MC-LR are 1) IFP 36 (Fig. 6a) located at the beginning of the simulation, 2) IFP 497 (Fig. 6b) and 3) IFP 507 (Fig. 6c) located at the end of the simulation. Some known interactions (Trp206, HOH) described in literature [46] were observed for all IFPs, others only for IFP 36 (His125, Tyr272, Cys273, Gly274) or for IFP 497 and IFP 507 (Asn124, Ile130, Cys127 (IFP507)), whereas some (Ile133, His125, Tyr134) were not found for any of the three major IFPs. Although the interactions with known residues are not necessarily of the same type as described in literature, the important residues and interactions have been identified, but never occur all together in the same IFP. In addition, we could identify Phe276, Val223 and Gln249 as important residues that were not identified in literature so far and might be relevant for further studies and evaluation. Phe276 and Val223 could also be identified with the aggregated_occ30_ frame proposed by Bouysset and Fiorucci [18] (see Additional file 1: Table S1), but Gln249 was not detected, highlighting the importance of analysing individual networks as they occur over a period of time. For MC-LF a similar trend is observed and the most frequent IFP patterns are shown in the appendix (see Additional file 1: Fig. S1b–d).

#### IFP comparison between MC congeners simulation

The IFP sets of MC-LR and MC-LF were merged to compare them and similarity evaluated based on the inverse Rogers-Tanimoto dissimilarity. A small proportion of identical IFPs (0.58%, 865 IFPs) could be identified between both MC congeners, 18.12% (or 26913 IFPs) were identified as similar, and 85.89% (127550 IFPs) as dissimilar. Please note that the numbers do not add up to 100% because one IFP can belong to several similarity classes depending on the reference IFP used for similarity calculation, therefore the values reflect the total number of comparisons in a data set.

**Fig. 7:**
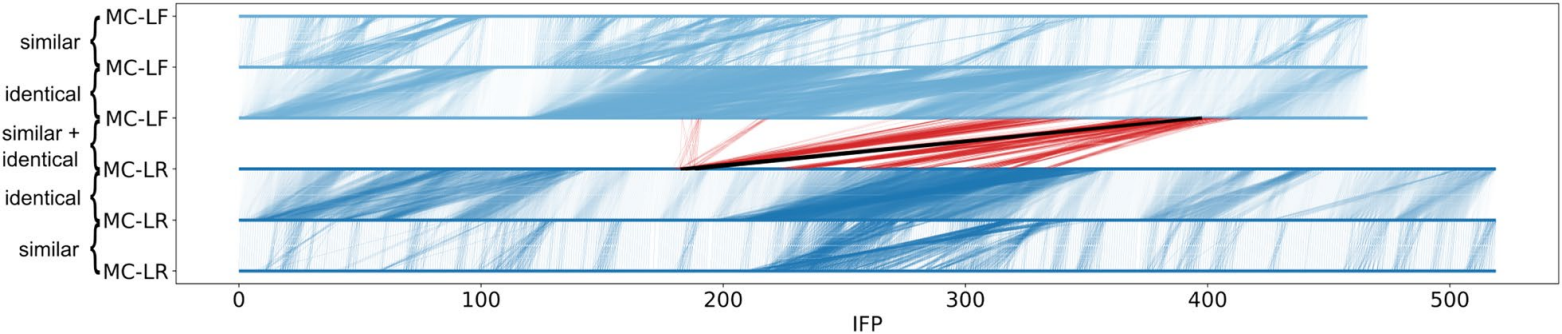
Comparison of IFP between MC-LR and MC-LF simulations. The first MC congener is shown in dark blue, the second in light blue. Identical and similar IFPs within the same simulation are indicated by vertical connections in the corresponding blue colour. Identical and similar IFPs between different simulations are shown as black and red lines respectively

Based on the number of IFPs in the different similarity classes, we conclude that while toxic MC congeners share some binding patterns, they also have distinct binding patterns which are specific to the respective MC congener. Figure 7 shows identical and similar IFPs of **MC-LR and MC-LF**. Similar and identical IFPs are connected by red or black vertical lines, respectively. IFPs of MC-LR map back to approximately four larger areas in MC-LF, which shows that both MC congeners share certain binding patterns, which confirms our initial hypothesis. The two MC congeners do not share too many binding patterns, as the majority of IFPs are not linked.

**Fig. 8:**
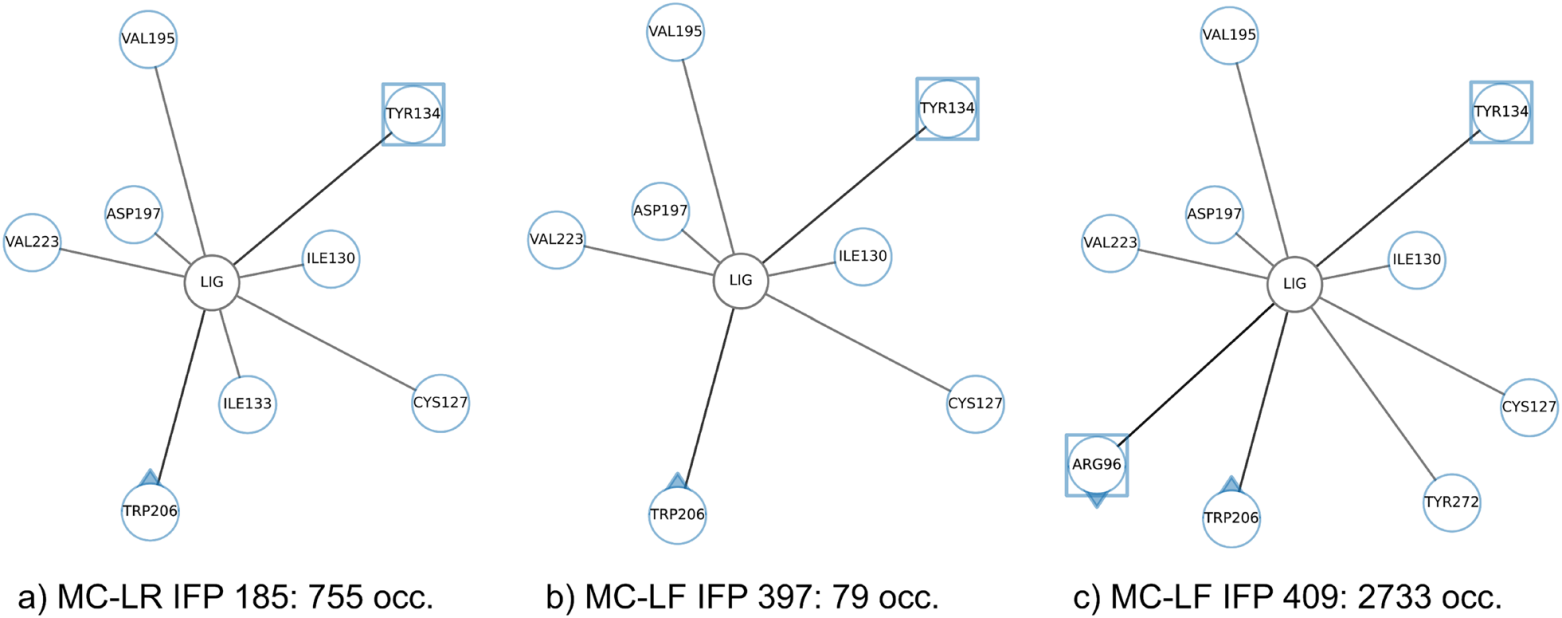
Comparison of IFPs between MC-LR and MC-LF simulations. The most frequent identical (**a**, **b**) and similar (**a**, **c**) IFPs of MC-LR and MC-LF are visualised as networks

In Fig. 8a–c, a set of identical and similar IFPs of both MC congeners that map to each other were selected based on the majority of occurrences. The two identical IFPs occurs 755 times in MC-LR (IFP 185, see Fig. 8a) and 79 times in MC-LF (IFP 397, see Fig. 8b). Both IFPs differ in only one interaction (Ile133 in MC-LR) demonstrating that our similarity threshold for identical IFPs is suitable to detect common IFPs across MC congeners and therefore across MD simulation data sets. Ile130, Ile133, Tyr134 and Trp206 are known in literature and could be efficiently retrieved. In comparison to the aggregated_occ30_ IFP not all interactions were retrieved for both MC congeners. Interestingly, Ile133 is more frequently occurring for MC-LF, although it was identified here for MC-LR which was not even detected in the aggregated_occ30_ IFP.

The two similar IFPs is also IFP 185 of MC-LR (occurrence 755, see Fig. 8a) and IFP409 in MC-LF (occurrence 2733, see Fig. 8c). Both share an overall interaction pattern with a difference in two protein residues (Arg96, Tyr272) and four interactions, indicating a good threshold chosen for similar IFP. Again, important residues for binding were identified that are described in literature: Cys127, Ile130, Ile133, Tyr134, Trp206 (both MC congeners), and Arg96 and Tyr272 for MC-LF. Interestingly, also here the aggregated_occ30_ IFP misses some interactions (e.g., Ile133 for MC-LR) even though this interaction should be retrieved for MC-LF, but not for this frequently occurring IFP (see Fig. 8c) identified here.

## Conclusion

Here we presented IFPAggVis, a library for systematic aggregation and comparison of IFPs to reduce the number of IFPs derived from MD simulations. The visualisations provide an overview to analyse simulations to derive biological knowledge and temporal development of interactions during simulation. We were able to identify representative IFPs based on our example data. Moreover, our aggregation method has the advantage of representing more realistic networks and analyse specific differences, since non-covalent interactions can form and break. Our analysis showed that we could reproduce known interacting residues from literature, which do never occur together in our representative IFPs. In addition, we were able to show that the aggregated_occ30_ IFP is a valid approach for a quick overview of IFPs, but suffers from missing interactions that occur frequently in individual IFPs and are therefore likely to be important. Moreover, we provide an estimate and visualisation to compare IFPs derived from different MD simulations and help to assess similarity of IFPs. IFPAggVis is a first step towards aggregation and comparison of IFPs derived from MD simulations and can be easily applied to other systems. Therefore, there are many possibilities for future developments. Currently, interactions are analysed at the residue level and the ligand is treated as a single entity. Incorporating interaction-based analysis at the atomic level could facilitate comparison between IFPs from different MD simulations. In addition, this approach could help to include or exclude certain atomic groups of the ligand that are of particular interest to the user. Moreover, the inclusion of interaction analysis in the 3D view could help to facilitate the analysis of interactions. From MD simulations, we can derive the 3D coordinates, but it is currently difficult to map the individual selected IFPs back to the respective frame of the MD simulation trajectory. Therefore, we want to include an automatic mapping of the IFPs to the frame of the MD simulation trajectory to improve the understanding of the interaction by including a 3D representation of the interacting molecules. In addition, the next step is to incorporate atom-based analysis, which will allow the inclusion and exclusion of specific chemical groups and could lead to improved analysis of interactions and comparison of IFPs between different MD simulations. Although we were able to massively aggregate the number of IFPs derived from the MD simulation, we believe that it is still possible to further aggregate and reduce the number of IFPs to a few representatives. These representatives could be analysed in more detail to improve our understanding of interaction and binding, or used for machine learning approaches.

### Supplementary Information

**Additional file 1. Table S1**: Aggregated_occ30_ IFP for MC-LR and MC-LF derived with ProLIF [18]. **Fig. S1**: Comparison of IFP similarity within MC-LF and most frequent IFP visualised as networks. **Fig. S2**: Zoomed in figure of number of IFPs (**a**, **b**) and interactions (**c**, **d**) after x_1_ and x_2_ filters are applied on MC-LR and MC-LF dataset.

## Data Availability

All the scripts and data used in this paper are available open source at Zenodo. [67, 68]. IFPAggVis as library is published on Github and tutorials are available to explain a typical workflow and usage. Project name: IFPAggVis. Project home page: https://github.com/LSI-UniKonstanz/IFPAggVis. Operating system(s): Platform independent. Programming language: Python. Other requirements: Python 3.8 or higher, and several open-source Python packages: ProLIF, MDAnalysis, RDKit, NumPy, tqdm, pandas, scikit-learn, Matplotlib, imageio, Networkx, DyNetx. License: Apache License 2.0. Any restrictions to use by non-academics: None.
