# Supplementary material for: Systematic analysis, aggregation and visualisation of interaction fingerprints for molecular dynamics simulation data
Source: J Cheminform. 2024 Mar 12;16:28. doi: 10.1186/s13321-024-00822-3 (PMC10935884; doi:10.1186/s13321-024-00822-3)
Supplement: Supplementary file 1 — Additional file 1. Table S1: Aggregatedocc30 IFP for MC-LR and MC-LF derived with ProLIF [18]. Fig. S1: Comparison of IFP similarity within MC-LF and most frequent IFP visualised as networks. Fig. S2: Zoomed in figure of number of IFPs (a, b) and interactions (c, d) after x1 and x2 filters are applied on MC-LR and MC-LF dataset. [file 13321_2024_822_MOESM1_ESM.pdf]

Supplementary Information: Systematic Analysis, Aggregation and Visualisation of Interaction Fingerprints for Molecular Dynamics Simulation Data

Sabrina Jaeger-Honz[1], Karsten Klein[1], Falk Schreiber[1,2]

[1]Department of Computer and Information Science, University of Konstanz, Konstanz
[2]Faculty of Information Technology, Monash University, Clayton

Table S1: The aggregated$_{occ30}$ IFP for MC-LR and MC-LF derived with ProLIF [1].

Interaction	MC-LR	MC-LF
HOH8 HBAcceptor	0.62	-
ARG96 HBAcceptor	0.35	0.55
ARG96 Anionic	0.38	0.59
HIS125 Hydrophobic	-	0.33
CYS127 Hydrophobic	0.35	0.38
ILE130 Hydrophobic	0.86	0.99
ILE133 Hydrophobic	-	0.38
TYR134 HBAcceptor	-	0.59
TYR134 Hydrophobic	0.33	0.56
VAL195 Hydrophobic	0.47	0.50
ASP197 Hydrophobic	0.43	0.43
TRP206 Hydrophobic	0.91	0.73
TRP206 PiStacking	-	0.39
ARG221 Hydrophobic	-	0.33
VAL223 Hydrophobic	0.74	0.58
HIS248 Hydrophobic	-	0.40
VAL250 Hydrophobic	0.59	0.71
VAL250 HBAcceptor	-	0.31
TYR272 Hydrophobic	0.60	-
TYR272 HBAcceptor	0.47	-
PHE276 Hydrophobic	0.36	
MN400 VdWContact	-	0.34
MN401 VdWContact	-	0.67
HOH402 HBAcceptor	-	0.35

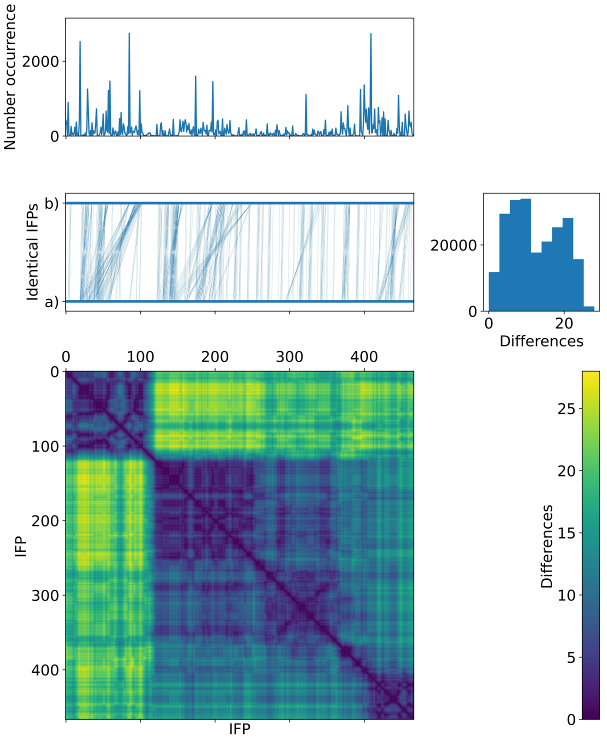

a) Number of differences and occurrences

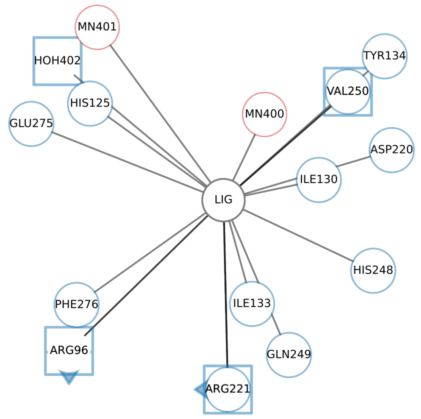

b) IFP 19: 2520 occurrences

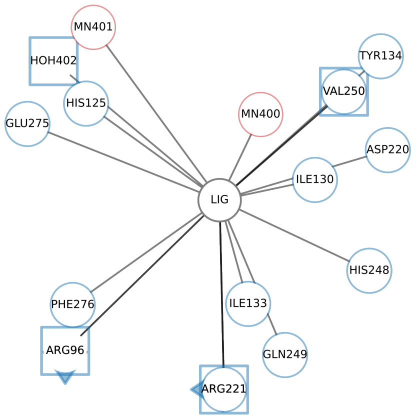

c) IFP 85: 2740 occurrences

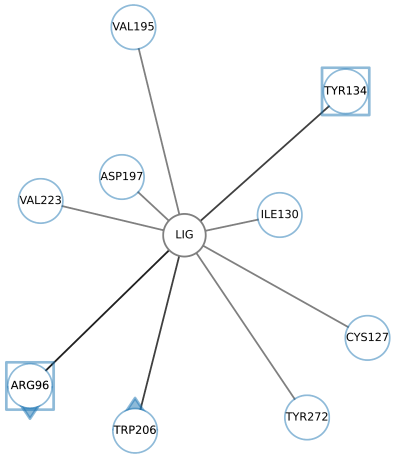

d) IFP 409: 2733 occurrences

Figure S1: Comparison of IFP similarity within MC-LF. a) shows the differences (colour on matrix visualisation, histogram (top right)) between IFP, identical IFP (line plot with identical IFP connected by vertical lines) and their occurrence (line plot at the top). In b), c) and d) the most frequent IFPs are visualised as networks.

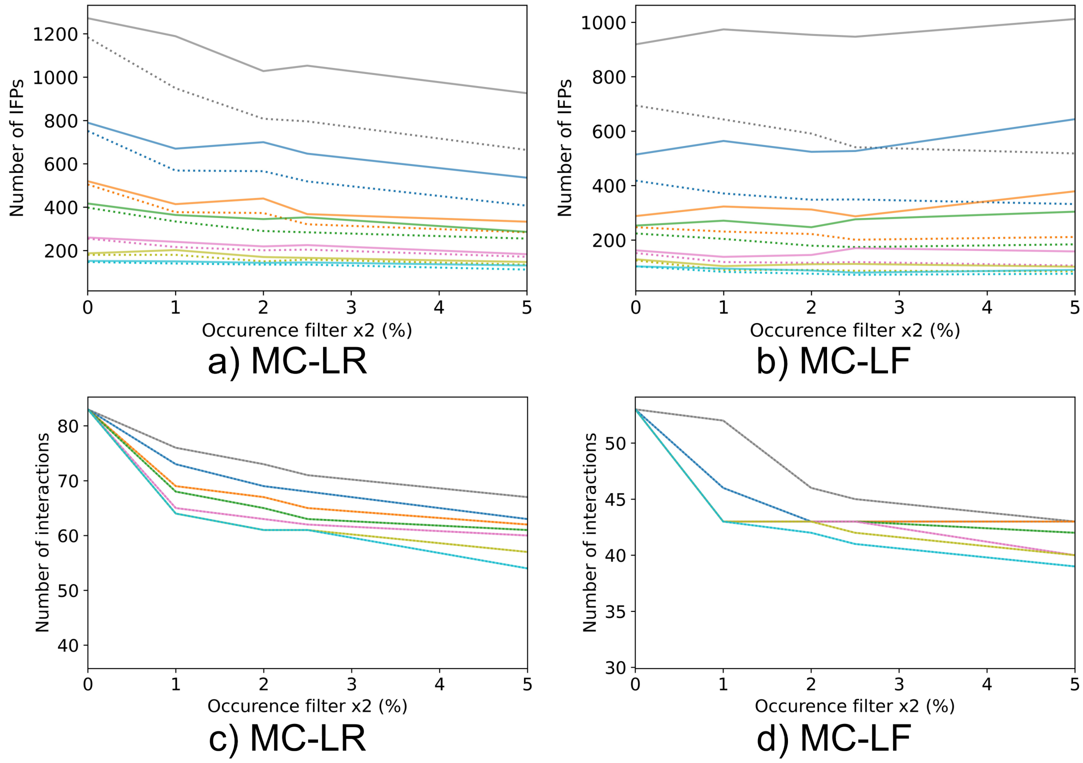

Figure S2: Number of IFPs (a and b) and interactions (c and d) after x_1 and x_2 filters are applied on MC-LR and MC-LF dataset. The x-axis shows the x_2 filter values from 0% to 5%, i.e. the figures are zoomed in. The x_1 filters are coloured by value: 0.5% is grey, 1% is blue, 2% is orange, 2.5% is green, 5% is pink, 7.5% is yellow and 10% is cyan. Solid and dashed lines represent aggregation based on time and interaction, respectively. The number of interactions is not affected by aggregation. Therefore, both lines are superimposed.

References

[1] Cédric Bouysset and Sébastien Fiorucci. ProLIF: a library to encode molecular interactions as fingerprints. *J. Cheminformatics*, 13(1):72, 2021.
